# Nano-Silica-Modified Chitosan-Based Membranes for Application in Direct Methanol Fuel Cells

**DOI:** 10.3390/polym17243281

**Published:** 2025-12-11

**Authors:** Livhuwani Elsie Modau, Tebogo Mashola, Rudzani Annetjie Sigwadi, Touhami Mokrani, Fulufhelo Nemavhola

**Affiliations:** 1 Department of Chemical Engineering, University of South Africa, Florida 1710, South Africa; 12739634@mylife.unisa.ac.za (L.E.M.);; 2 Department of Mechanical Engineering, Faculty of Engineering and the Built Environment, Durban University of Technology, Durban 4000, South Africa; 3 College of Graduate Studies, University of South Africa, Pretoria 0001, South Africa

**Keywords:** nanocomposite, silica, chitosan, membrane, fuel cell

## Abstract

Membrane electrolytes play a critical role in energy conversion devices. The development of stable, efficient membrane electrolytes is urgent and demands paramount attention for the successful commercialization of fuel cells. Chitosan, a naturally occurring material, and silica particles were used as precursors for organic–inorganic membrane polymers. The silica nanoparticles were prepared by the sol–gel and Stober methods and characterized using various techniques, including XRD, FTIR, etc. The silica-incorporated membranes show improved properties, with the sulfur-functionalized membranes having optimal proton conductivity, ion-exchange capacity, and tensile strength of 0.0238 S/cm, 2.86 meq/g, and 7.3 MPa, respectively. It also showed the lowest methanol permeability. This was clear proof that membrane functionalization has a positive impact on tuning the properties of electrolyte membranes and should be further explored in membrane development.

## 1. Introduction

Direct methanol fuel cells (DMFCs) are a potential sustainable energy technology for both portable and stationary power generation [1]. The membrane plays a crucial role in the performance of these cells. Hence, several membranes such as Nafion, sulfonated polyether ether ketone, and composites have been used in the application of DMFCs. Proton exchange membrane (PEM) Nafion has attracted attention due to its desirable ionic conductivity. However, it has considerable disadvantages, such as high methanol permeability, high manufacturing costs, and moisture-dependent proton conductivity, which call for an alternative. As a result, much research has focused on creating alternative, cost-effective PEM materials that preserve competitive performance while reducing fuel use. Chitosan has been explored as a substitute because it is cheap and readily available [1]. However, it has low ionic conductivity and high swelling properties that inhibit its application in fuel cells [2]. A lot of research has been performed to modify chitosan so that it can be employed in DMFCs. These processes include sulfonation, adding plasticizers and fillers such as silica (SiO_2_). Silica has been employed in improving properties of chitosan membranes and several reports showed promising results [3,4,5,6]. The essential distinction is between the synthesis procedures used to create silica particles and their subsequent surface modification. However, the effects of various production processes on membrane functionality have not been thoroughly investigated. The shape and the development of effective membranes synthesized using different processes may differ, which may influence their dispersion in chitosan and total proton transfer. Furthermore, modification of silica through sulfonation can introduce extra acid sites, increasing conductivity. This project further explored silica-based nanoparticles as dopants, aiming of improving the conductivity of a Nafion nanocomposite membrane.. In situ sol–gel synthesis has been demonstrated to produce better dispersions than direct addition of pre-synthesized nanoparticles, resulting in improved membrane characteristics [2]. Additionally, it was reported that the difference between pure and sulfonated silica reflects a fundamental shift in composite membrane design philosophy. Pure silica (SiO_2_) improves hydrophilicity with hydroxyl groups, leading to better water retention and reduced methanol permeability [7]. An earlier study by Pannipara et al. [8] found that chitosan/sulfonated-graphene-oxide/silica hybrid membranes have improved proton transport to 10^−2^–10^−1^ S/cm depending on temperature and hydration. According to Doobi [9], well-engineered chitosan–silica membranes have proton conductivities ranging from 10^−3^ to lower 10^−1^ S/cm. The highest values are seen when sulfonic groups are introduced, and sufficient hydration is maintained. The diffusion-blocking effect of silica domains reduces methanol permeability to 10^−7^–10^−6^ cm^2^/s. Tensile strength stays in the few-MPa range and improves marginally at low filler concentrations around 1–2 wt% [9]. These experiments show that low-sulfonated silica loadings enable uniform dispersion and offer the best balance of conductivity, methanol resistance, and mechanical stability [9]. Thus, chitosan has proven to be a promising natural polymer for effective membrane development. Hence, it was explored further in this project using silica-based nanoparticles as the dopants with the aim of developing an effective membrane with long-term stability for fuel cells.

## 2. Materials and Methods

### 2.1. Materials

All solutions were prepared with deionized water. All the chemicals used were purchased from Merck (Sigma-Aldrich (PTY) Ltd., Pomona AH, Gauteng, South Africa) and were of analytical grade. The chemicals employed comprised chitosan flakes (medium molecular weight, 98%), tetraethyl orthosilicate (Si(OC_2_H_5_)_4_), acetic acid (CH_3_COOH, 99%), ammonia solution (NH_3_, 25%), sodium hydroxide (NaOH), ethanol (C_2_H_5_OH, 99.9%), methanol (CH_3_OH, 99.9%), sodium chloride (NaCl), hydrochloric acid (HCl, 37%), sulfuric acid (H_2_SO_4_, 99%), and taurine (C_2_H_7_NO_3_S, 99%).

### 2.2. Methodology

#### 2.2.1. Synthesis of Unmodified Silica Particles by Sol–Gel Method

The synthesis of silica was performed using the sol–gel and Stöber methods to evaluate whether the synthesis method affects the properties of silica and the membrane. About 80 mL of tetraethyl orthosilicate (TEOS) and ethanol (200 mL) were mixed at ambient temperature under stirring for 30 min (solution A). Solution B, an ammonium and water solution, was then added to solution A, and the mixture was agitated for 1 h at 70 °C. A whitish gel-like substance was obtained and dried at 100 °C for 24 h. The obtained silica particles were calcinated at 600 °C for 24 h to remove volatile substances such as water from the silica.

#### 2.2.2. Synthesis of Unmodified Silica Particles by the Stober Method

Solution A was prepared as stated on Section 2.2.1 and was mixed with DI water. Afterwards, ammonia was introduced into solution A dropwise, and the mixture was agitated for 1 h at ambient temperature. The resultant solution was centrifuged for 31 min 44 s at 3700 rpm. The obtained white solid gel, namely silica, was dried at 100 °C for 24 h and calcinated at 600 °C.

#### 2.2.3. Sulfonation of Silica Particles

Sulfonation of the prepared silica was performed using sulfuric acid to enhance the proton conductivity of the particles. Both particles, prepared using the sol–gel and Stober processes, underwent the same sulfonation process. About 10 g of silica particles was sulfonated with 200 mL of sulfuric acid under constant stirring at 1500 rpm for 4 h. The solution was centrifuged for 31 min 44 s to obtain sulfonated silica particles.

#### 2.2.4. Membrane Fabrication and Casting

About 5 g of chitosan powder was mixed with acetic acid to make 2% *v*/*v* solution (solution A) which was stirred for 1 h. In another beaker, silica particles (0%, 2%, 4%) and 0.75 g of taurine (functionalizing agent) were mixed with DMSO to make a second solution (solution B). Solution B was gradually added to solution A under continuous stirring. The resulting mixture was cast into a Petri dish and subsequently dried. The obtained membrane was rinsed with deionized water to remove any residual acid from the surface. The resulting suspension was dried for 5 h at 70 °C. Sulfonated silica–chitosan membranes were also made using this process.

## 3. Results

### 3.1. Fourier Transform Spectroscopy (FTIR) Analysis

**Figure 1:**
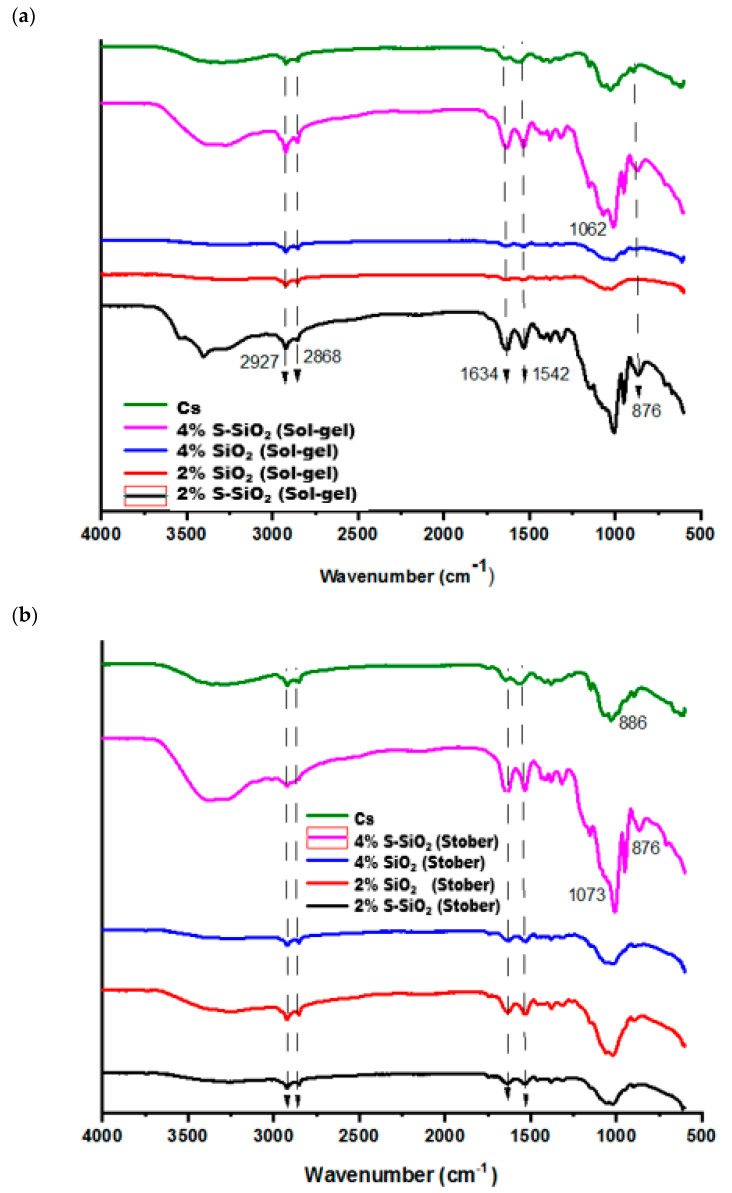

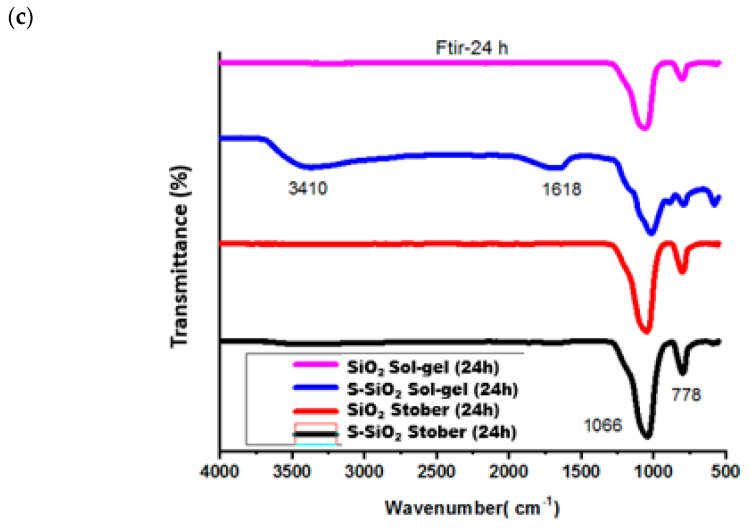
FTIR spectra of Cs, 4% s-SiO_2_/Cs, 4% SiO_2_/Cs, 2% SiO_2_ and 2% s-SiO_2_ prepared by (**a**) sol-gel, (**b**) Stober methods, and (**c**) pristine and sulfonated silica prepared by sol-gel and Stober meth-ds.

In Figure 1, FTIR was used determine functional groups of the chitosan-based membranes using a Fourier-transform infrared (FTIR) spectroscopy (IR Tracer-100, Shimadzu, Kyoto, Japan) within the wavenumber range of 500–4000 cm^−1^. Figure 1a shows the FTIR spectra of chitosan membranes that contain sulfonated silica particles and pure SiO_2_ at 0%, 2%, and 4%. The detected -OH stretching bands at 3429 cm^−1^ indicate the existence of hydroxyl groups, suggesting their role in facilitating proton conduction within the membrane matrix in the FTIR spectra of the chitosan. The N-H_2_ deformation vibrations on the raw chitosan membrane are directly correlated with the band at 1542 cm^−1^, while an absorption band observed at 1420 cm^−1^ corresponds to the C-H bending vibrations, confirming the presence of aliphatic hydrogen atoms in the structure. Also, the C-H was responsible for the bending vibrations at 1380 cm^−1^. The absorption band at 1309 cm^−1^ is attributed to the asymmetric stretching vibrations of the C-O-C linkage, while Si-O-Si exhibits an asymmetric stretching vibration at 1012 cm^−1^ [7]. An enhancement in the intensity of the band at 850–1200 cm^−1^ on the silica-modified chitosan is caused by overlapping of the IR peak caused by the twisting of the silica on the chitosan matrix. The shoulders that show up at about 800 cm^−1^ on the chitosan modified matrix indicate the formation of the Si-OH network. The FTIR spectrum in Figure 1b had the same functional group as the fabricated membranes in Figure 1a but was not located at the same number of waves due to the interaction of the chitosan with the incorporated silica particles. Functional group changes have been observed on both pure and sulfonated FTIR spectra of silicon-modified chitosan, which indicates successful modification. The symmetric S=O stretching coincides with the Si-O-Si band at 1030–1080 cm^−1^, resulting in significantly increased intensity in this zone. Both spectra (particularly the s-SiO_2_) show a significant shoulder between 880 and 890 cm^−1^, indicating successful S-O stretching of grafted -SO_3_H groups.

### 3.2. X-Ray Diffraction (XRD)

**Figure 2:**
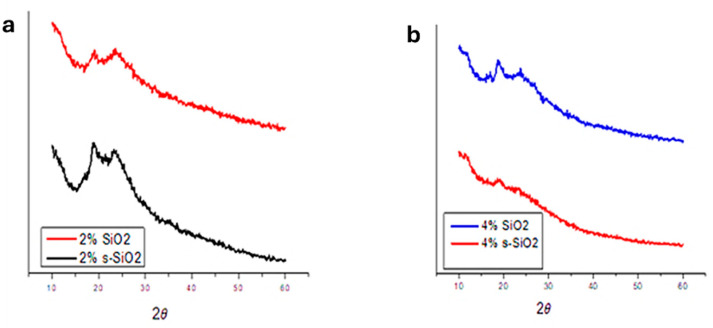
XRD spectra of (**a**) 2% and (**b**) 4% silica-modified chitosan membranes prepared by the sol–gel method.

**Figure 3:**
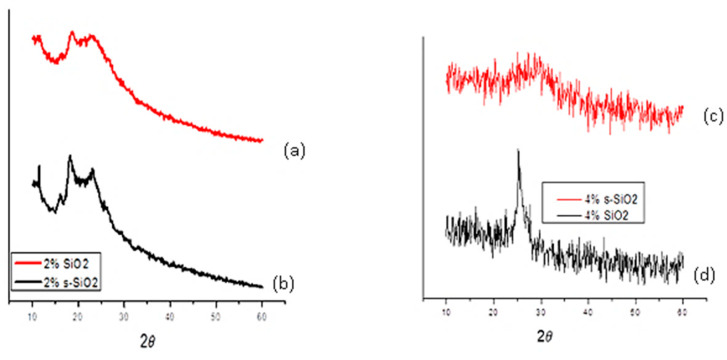
XRD spectra of (**a**) 2% SiO_2_, (**b**) 2% s-SIO_2_, (**c**) 4% s-SiO_2_ and (**d**) 4% SiO_2_ silica-modified chitosan membranes prepared by the Stober method.

Figure 2 and Figure 3 illustrate the XRD pattern of chitosan membranes modified with 2% and 4% SiO_2_/s-SiO_2_. It was observed that all composite membranes retain amorphous profiles, with noticeable differences depending on silica quantity and synthesis process. Membranes with silica synthesized via sol–gel exhibit a broad peak near 2θ = 20–25°, which widens and becomes less prominent as the amount of silica increases, indicating greater disruption of the chitosan chains. This may be because the silanol groups in silica form hydrogen bonds with the -OH and -NH_2_ groups in chitosan, creating a more amorphous and chemically connected hybrid network. Furthermore, silica particles become embedded in the polymer matrix, inhibiting chain mobility and the creation of crystalline microstructures [10,11]. However, 4% SiO_2_ and 4% s-SiO_2_ membranes prepared by the Stober method exhibit a more amorphous properties, with diffraction peaks located at 24°. The amorphous nature of materials prepared by Stober method compared to sol–gel method can be attributed to the faster condensation, controllable kinetics, nucleation and growth which result in small uniform nanoparticles. The s-SiO_2_ chitosan membrane is known to display a more amorphous character relative to SiO_2_ because of the partial reconstruction of the SiO_2_ crystal regions during the transformation process [12,13]. The results are an indication that silica was able to successfully interact with the chitosan matrix as it is known to be semi-crystalline in nature [14,15].

### 3.3. Scanning Electron Microscopy (SEM)

**Figure 4:**
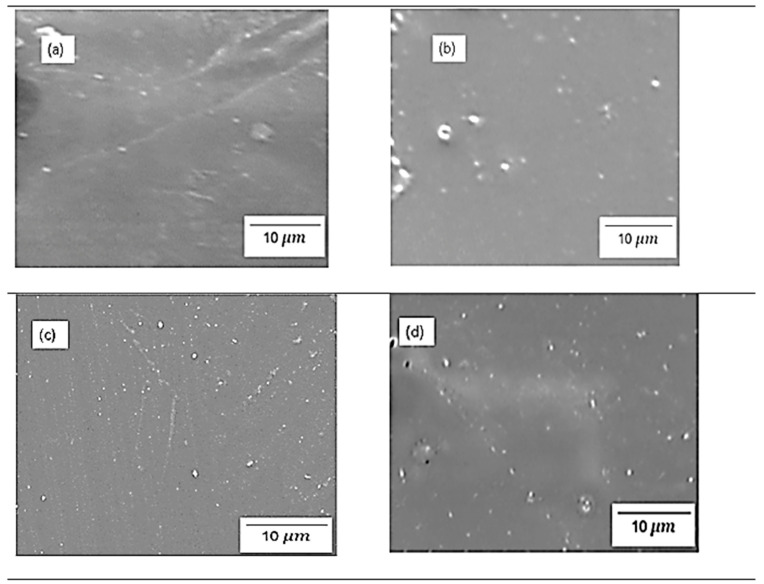
SEM images of chitosan incorporated with (**a**) 2% SiO_2_ (**b**) 2% s-SiO_2_ (**c**) 4% SiO_2_ (**d**) 4% s-SiO_2,_ prepared by the sol–gel method.

**Figure 5:**
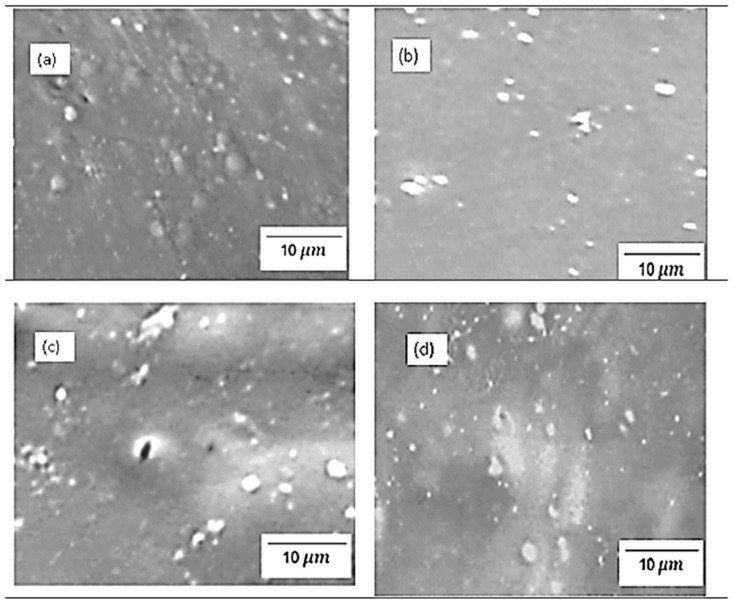
SEM images of chitosan incorporated with (**a**) 2% SiO_2_ (**b**) 2% s-SiO_2_ (**c**) 4% SiO_2_ (**d**) 4% s-SiO_2,_ prepared by the Stober method.

Figure 4 and Figure 5 show the physical morphology of chitosan membranes incorporated with 2% SiO_2_, 2% s-SiO_2_, 4% SiO_2_, and 4% s-SiO_2_ particles. The presence of silica in the membrane is denoted by white particles embedded in the chitosan matrix. At lower silica loading of 2%, very few silica particles can be seen on the membranes, and these particles are sparsely dispersed and well-integrated, particularly in Figure 4, indicating high compatibility via hydrogen bonding between silanol groups and chitosan amine/hydroxyl groups. The 2 wt% of membranes in Figure 4 have smooth surfaces compared to the rough morphology seen in Figure 5a. The appearance of lumps in Figure 5a can be attributed to processing factors. For example, when dispersion is inadequate, or when silica condenses too quickly, the micro-clusters (agglomerates) become trapped within the chitosan matrix and appear as elevated surface features in SEM [16]. Also, the presence of bubbles during synthesis can cause this. However, at higher silica loading (4%), the particle population becomes denser, and small agglomerates appear. This morphological characteristic is comparable with those reported in the literature, where greater silica content frequently causes partial phase separation or micro-cluster formation because of condensation-driven network growth [14]. In contrast, membranes containing sulfonated silica have a more rough and diverse appearance. This is due to strong polarity of -SO_3_H groups that boost hydrophilicity and surface energy, resulting in more noticeable inorganic–organic interfaces. Also, functionalized silica, particularly sulfonated or grafted, forms bigger clusters due to greater intermolecular interactions and the creation of acid-rich microdomains [17]. Although the silica within the membranes displays evidence of aggregation, it is well-distributed across the membrane structure.

### 3.4. Water Uptake Analysis of Pure and Sulfonated Chitosan Membranes with Silica

#### 3.4.1. Effect of Silica Content on Water Uptake

**Figure 6:**
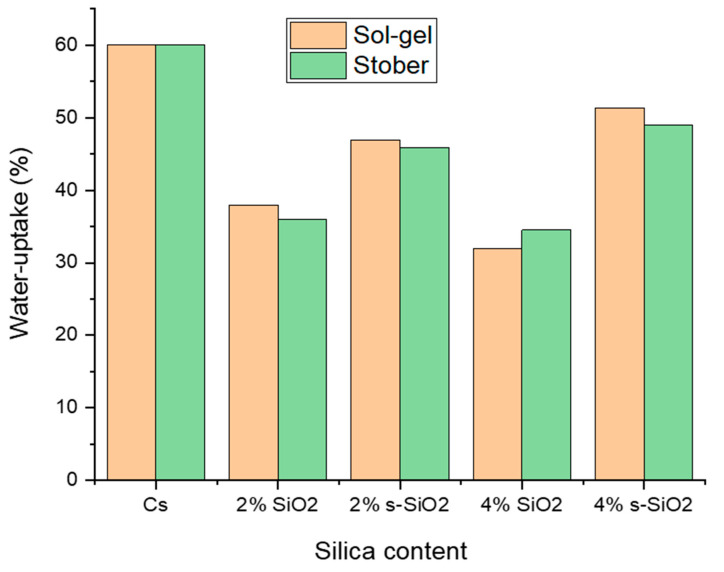
Water uptake of chitosan membranes incorporated with silica nanoparticles prepared using sol–gel and Stober methods.

Figure 6 shows the water absorption rate of the chitosan membrane at ambient temperature. The results indicate that water absorption of silica-incorporated chitosan membranes decreases with an increase in silica content for the non-functionalized silica. However, on membranes with modified silica, it was observed that when the amount of silica added in the membrane increases, the water absorption of the membrane also increases. This occurs because non-functionalized silica acts as a physical filler, reducing membrane pore volume and interfering with water permeation pathways [18]. Contrarily, modified silica adds Si-OH (silanol) and Si-O-Si (siloxane) groups that form hydrogen bonds with water molecules. These groups are naturally hydrophilic, which promotes water uptake [19]. This is also supported by the findings in this study as chitosan membranes incorporated with functionalized silica nanoparticles showed high water uptake capacity compared to the chitosan incorporated with non-functionalized silica nanoparticles. The optimum uptake reported is 56.21% which is ascribed to 4% s-SiO_2_ of the sol–gel, followed by 4% s-SiO_2_ of the Stober method with an uptake of 48.92%. Comparable results were reported in the literature exhibiting a water uptake of approximately 62% for 4% SiO_2_/Cs membrane, which is slightly higher than the one found in this study [3]. Despite modification, the membranes show an increase in water uptake as the filler content increases. However, although increases were observed, the membranes were still able to inhibit water absorption at a lower rate than 60% of pure chitosan.

#### 3.4.2. Effect of Temperature on Water Uptake

**Figure 7:**
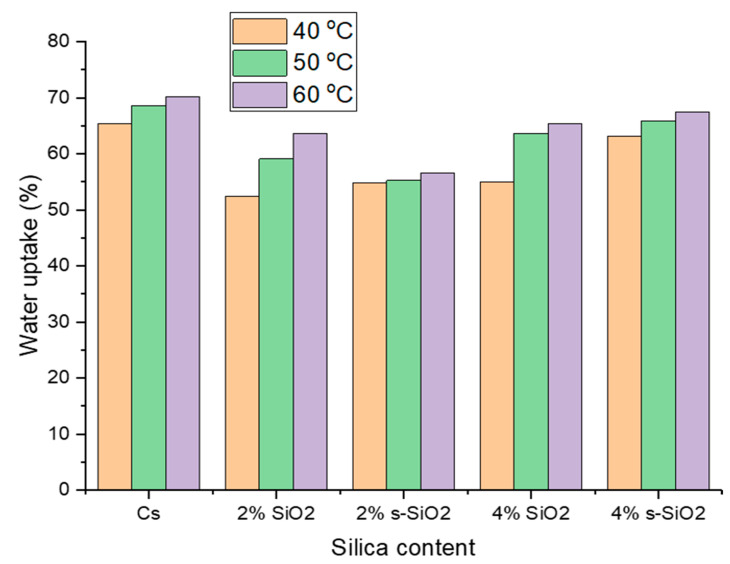
Effect of temperature on the water uptake of chitosan membranes. (Silica nanoparticles prepared with the sol–gel method).

**Figure 8:**
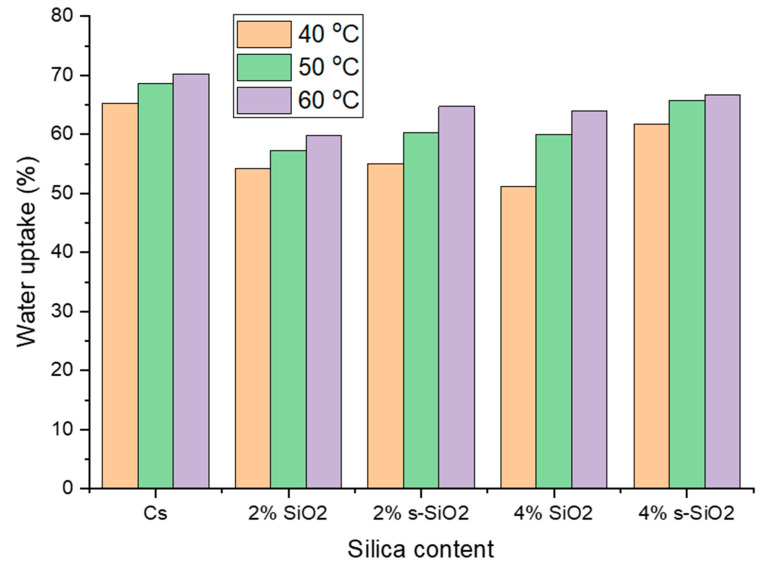
Effect of temperature on the water uptake of chitosan membranes. (Silica nanoparticles prepared with the Stober method).

Figure 7 and Figure 8 indicate the influence of temperature on raw and silica-modified chitosan membranes at temperatures of 40 °C, 50 °C, and 60 °C. As seen in Figure 7 and Figure 8, the membranes’ water uptake increases with increasing temperature, showing a direct positive relationship between temperature and hydration. This can be attributed to increased kinetic energy of water due to external energy supply, resulting in fast and free movement of water at high temperatures compared to lower temperatures. At increasing temperatures, water molecules and chitosan polymer chains become more mobile, allowing them to pass through the membrane. Furthermore, the inclusion of silica silanol groups increases the hydrophilicity of chitosan, which promotes water absorption at high temperatures [20,21]. Similar results were reported by Tsen et al. [22].

### 3.5. Ion-Exchange Capacity (IEC) Analysis

**Figure 9:**
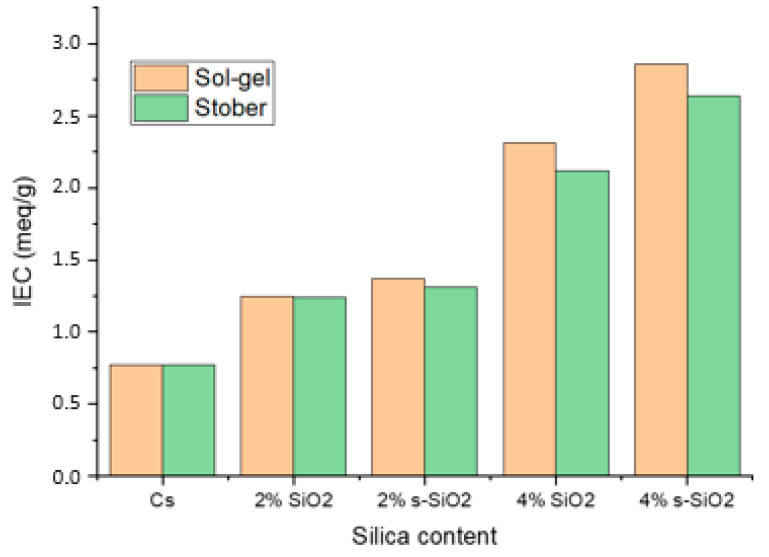
Ion-exchange capacity of chitosan and chitosan modified with 2% SiO_2_, 2% s-SiO_2_, 4% SiO_2_ and 4% s-SiO_2_ nanoparticle prepared by the sol–gel and Strober method.

Figure 9 represents the ion-exchange capacity of raw modified silica. The IEC values were determined theoretically based on the number of sulfonic acid groups in the polymer, and they were subsequently tested using the titrimetric method. The following equation was used to determine (IEC):

$$IEC=\frac{∆V\times C}{W_{d}}$$

where ∆V is the volume consumed, C is the concentration of the solution and W_d_ is the mass of the dehydrated membrane. In Figure 9,an increase in IEC is observed upon silica modification on the matrix of pure chitosan. Pure chitosan has an IEC of 0.77 meq/g, which increased to 2.86 and 2.64 meq/g upon modification of chitosan with 4% s-SiO_2_ of sol–gel and Stober method, respectively. It is noticeable that the highest ion-exchange capacity is reported in composite membranes with functionalized silica. The rise in IEC is caused by silica, which forms a close bond with chitosan, and the sulfate ions in the polymer’s structure, which act as proton exchange sites and establish conditions that favor efficient ion exchange within the membrane matrix [23]. Membranes containing silica nanoparticles generated via the sol–gel method exhibit greater IEC values compared to those modified with silica derived via the Stober process, highlighting the importance of the synthesis route on ion-exchange capacity.

### 3.6. Proton Conductivity Analysis

**Figure 10:**
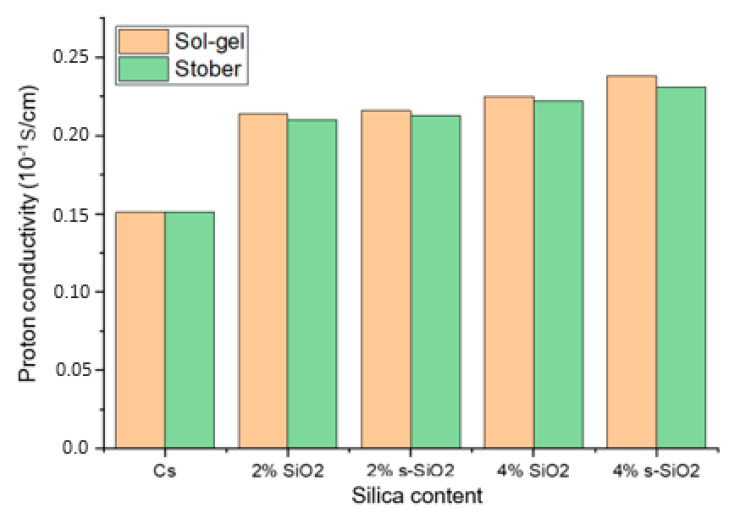
Proton conductivity of chitosan and chitosan modified with 2% SiO_2_, 2% s-SiO_2_, 4% SiO_2_ and 4% s-SiO_2_ membranes.

Figure 10 shows the proton conductivity of the chitosan-based composite membranes. The proton conductivity test was conducted at a fully hydrated state of chitosan (100% RH). The findings in Figure 10 show that the proton conductivity of modified membrane exceeds that of raw chitosan (0.0151 S/cm), which suggests that the modification increases the proton transport. Furthermore, the ionic conductivity increases as the amount of silica added increases, with 2% Cs/SiO_2_ or 2% Cs/s-SiO_2_ membranes having the smallest proton conductivity of 0.0214 and 0.021 S/cm and the highest proton conductivity of 0.0238 and 0.031 S/cm for sol–gel and Stober, respectively. In addition, the CS/s-SiO_2_ membranes exhibited the highest proton conductivity in contrast to CS/SiO_2_ membranes. The improved proton conduction can be attributed to the filler’s better dispersion and the structural advantages it brings to the membrane. Also, proton transport in chitosan involves hopping between -NH_2_ groups and adjacent -NH_3_^+^ sites. So, the addition of silica increases the proton diffusion pathway by providing additional bridging sites, allowing for more effective proton transfer between surrounding regions within the polymer matrix [1,9,24]. Additionally, the addition of s-SiO_2_ to these membranes serves as a passage to link them (s-SiO_2_) and shortens the proton hopping path length. Because of this, CS/s-SiO_2_ membranes were more conductive than CS/SiO_2_ membranes. On the other hand, the increase in proton conductivity at higher silica loading can be attributed to the increase in the mobility of water molecules and polymer chains in the membrane. Under these conditions, the Grotthuss mechanism becomes more effective because dissociated protons quickly bond with nearby water molecules and transmit via a continuous hydrogen-bond relay, allowing for faster and more efficient proton transport across the membrane. This can also be attributed to better silica dispersion shown in Figure 4 and Figure 5. The amorphous nature of the material reported in Figure 3 also played a vital role as it is reported that in polymer electrolytes; amorphous domains have a higher free volume and more flexible chain segments than crystalline regions. This structural looseness allows water molecules to move more freely and creates continuous hydrogen-bonded routes, both of which are required for efficient proton transport. And, when silica or sulfonated groups are introduced, they further disrupt the native semi-crystalline ordering of chitosan, providing a more disordered matrix that allows protons to hop more freely via the Grotthuss mechanism [25,26].

### 3.7. Methanol Permeability Analysis

**Figure 11:**
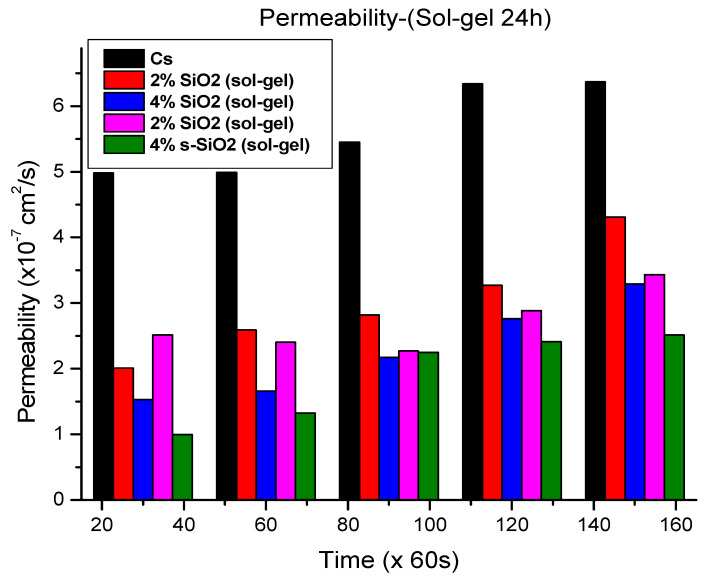
Methanol permeability of chitosan and chitosan modified with 2% SiO_2_, 2% s-SiO_2_, 4% SiO_2_ and 4% s-SiO_2_ membranes (silica particles prepared by sol–gel method).

**Figure 12:**
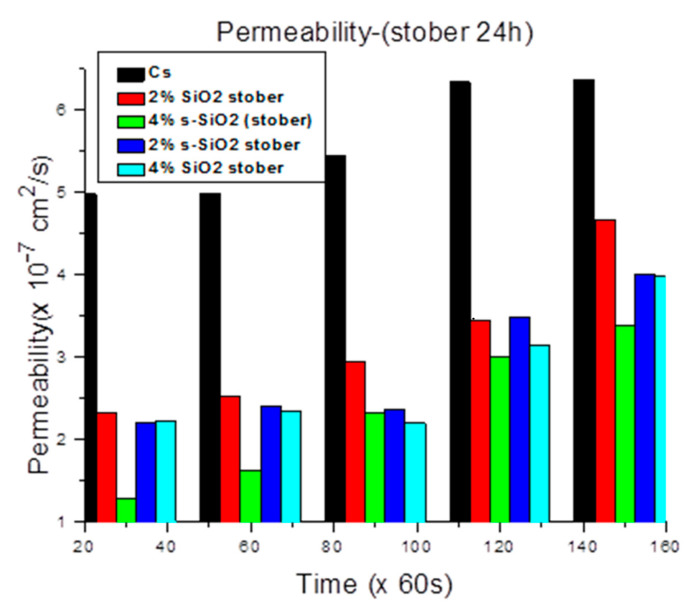
Methanol permeability of chitosan and chitosan modified with 2% SiO_2_, 2% s-SiO_2_, 4% SiO_2_ and 4% s-SiO_2_ membranes (silica particles prepared by Stober method).

Figure 11 and Figure 12 demonstrate that adding silica into chitosan considerably reduces methanol permeability. The sol–gel membranes in Figure 11 reduced methanol crossover from 4.98 × 10^−7^ cm^2^/s to 1.53 × 10^−7^ cm^2^/s and 0.995 × 10^−7^ cm^2^/s with 4% silica loading. In Figure 12, the Stober-derived membranes with 4% and 2% sulfonated silica have the lowest permeability values of 1.29 × 10^−7^ cm^2^/s and 2.217 × 10^−7^ cm^2^/s, respectively, at the 30 min interval. These results emphasize the substantial barrier effect generated by silica, which restricts the movement of methanol more efficiently at high silica quantity. It was reported that increasing the silica quantity improves the proton conductivity due to its ability to reshape the membrane’s microstructure for more selective transport. However, the interaction of chitosan with nano-silica creates a more tortuous pathway which leads to a decrease in methanol permeability. Even though protons can still travel easily through the interconnecting hydrophilic channels, larger methanol molecules find it more difficult to migrate over the membrane due to structural limitations. As a result, when silica loading increases, the membrane becomes more tightly packed, methanol suffers higher resistance, and proton mobility benefits from the improved hydration and continuous ionic channels [26,27,28,29]. It was also observed that methanol permeability increases with time. The time-dependent rise in methanol permeability reflects the progressive emergence of preferred transport routes within the membrane. Water and methanol mobility inside polymer membranes’ hydrophilic channels change over time as molecules form equilibrium distributions and interaction patterns with the polymer chains [30,31].

### 3.8. Tensile Strength Analysis

**Figure 13:**
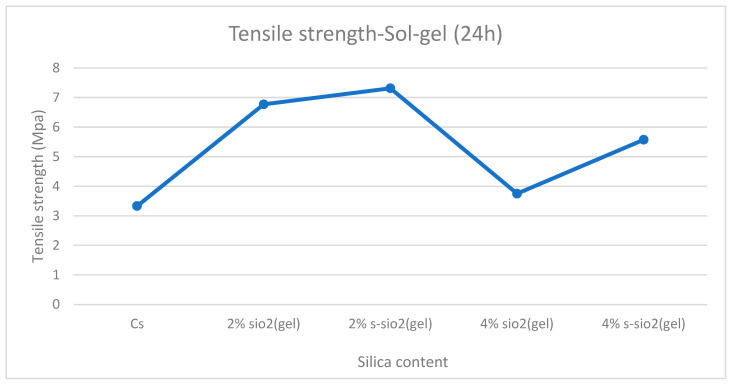
Tensile strength of chitosan and chitosan modified with 2% SiO_2_, 2% s-SiO_2_, 4% SiO_2_, and 4% s-SiO_2_ membranes (silica particles prepared by sol–gel method).

**Figure 14:**
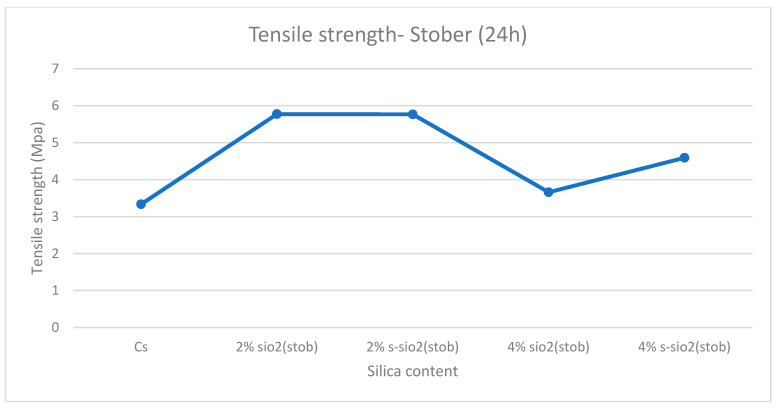
Tensile strength of chitosan and chitosan modified with 2% SiO_2_, 2% s-SiO_2_, 4% SiO_2_, and 4% s-SiO_2_ membranes (silica particles prepared by Stober method).

Figure 13 and Figure 14 demonstrate tensile strength of raw and modified chitosan membranes with silica synthesized by the sol–gel and Stober process. The tensile strength of the modified membranes ranges from 3.65 MPa to 7.3 MPa, whilst that of raw chitosan is at 3.33 MPa. The tensile data reveals that minor silica loadings of 2% increase the mechanical integrity of chitosan films, while higher loadings of 4% result in a significant loss in strength. The decrease can be due to high agglomeration and surface protrusions at high-silica-loaded membranes reported in Figure 4 and Figure 5. This is because the mechanical strength declines with increased silica loading because the polymer network is gradually disturbed by the excessive inorganic phase. Hence, if too much silica is added, these interactions are disrupted and the matrix loses rigidity. Also, the agglomeration of silica produces micro-defects that act as stress concentration points, increasing the membrane’s vulnerability to deformation. At the same time, the interfacial connection between chitosan and silica weakens, limiting effective stress transmission and making the filler behave more like a defect than a reinforcement. Similar results were also found by Film et al. [32].

## 4. Conclusions

The modification of chitosan membrane with fillers improves its chances of application in DMFC. However, different parameters play a crucial role for successful modification. Factors such as synthesis method, type of fillers, and different synthesis conditions need to be controlled for effective application in DMFC. The addition of silica significantly disturbs the semi-crystalline structure of chitosan, increasing its amorphous content and allowing for more efficient proton transport through segmental mobility and water retention. Sulfonated silica also contributes by adding new proton-donating sites and enhancing hydrogen-bonded networks inside the membranes. From the results it can be concluded that the Stober method is the best for silica nanoparticle preparation. Chitosan membranes modified with sulfonated silica showed the best performance compared to other membranes. However, more work needs to be performed to improve the performance of chitosan–silica membranes, by focusing on more systematic research methods required to determine the optimal loading range for both selectivity and durability of the membranes. The membranes have promising requisite properties for fuel cell application. Thus, future work will include the fuel cell performance test for these membranes.

## Data Availability

The original contributions presented in this study are included in the article. Further inquiries can be directed to the corresponding authors.
